# Trehalose Ameliorates Zebrafish Emotional and Social Deficits Caused by CLN8 Dysfunction

**DOI:** 10.3390/cells14010055

**Published:** 2025-01-05

**Authors:** Rosario Licitra, Stefania Della Vecchia, Lorenzo Santucci, Rachele Vivarelli, Sara Bernardi, Filippo M. Santorelli, Maria Marchese

**Affiliations:** 1 Neurobiology and Molecular Medicine Unit, IRCCS Fondazione Stella Maris, 56128 Calambrone, Italy; rosario.licitra@vet.unipi.it (R.L.); stefania.dellavecchia@fsm.unipi.it (S.D.V.); lore.santucc@gmail.com (L.S.); rachelevivarelli@gmail.com (R.V.); sara.bernardi@fsm.unipi.it (S.B.); filippo.santorelli@fsm.unipi.it (F.M.S.); 2 Department of Veterinary Sciences, University of Pisa, 56124 Pisa, Italy

**Keywords:** neuronal ceroid lipofuscinoses, CLN8, translational medicine, zebrafish, behavior, trehalose, dietary treatment

## Abstract

CLN8 and other neuronal ceroid lipofuscinoses (NCLs) often lead to cognitive decline, emotional disturbances, and social deficits, worsening with disease progression. Disrupted lysosomal pH, impaired autophagy, and defective dendritic arborization contribute to these symptoms. Using a *cln8^−/−^* zebrafish model, we identified significant impairments in locomotion, anxiety, and aggression, along with subtle deficits in social interactions, positioning zebrafish as a useful model for therapeutic studies in NCL. Our findings show that trehalose, an autophagy enhancer, ameliorates anxiety, and modestly improves social behavior and predator avoidance in mutant zebrafish. This finding aligns animal models with clinical reports suggestive of behavioral improvements in NCL patients. Trehalose holds promise as a therapeutic agent for CLN8, warranting further research into its neuroprotective mechanisms and clinical applications.

## 1. Introduction

Neuronal ceroid lipofuscinosis type 8 (CLN8) is a late-childhood-onset form of neuronal ceroid lipofuscinosis (NCLs), with an onset on average after 4 years of age, and it accounts for 2–3% of all NCLs [1]. The *CLN8* gene encodes for an endoplasmic reticulum (ER)-cargo receptor that regulates lysosome biogenesis [2] by transferring lysosomal enzymes from the ER to the Golgi apparatus. CLN8 loss of function impairs lysosomal storage [3], resulting in altered autophagy [4]. Previous work [5] demonstrated that CLN8 participates in vesicular distribution and maintenance of lysosomal pH. Moreover, CLN8 is essential for the development of the dendritic tree, which plays a fundamental role in neuron connectivity and communication. Proper dendritic growth and branching are necessary for normal synaptic function and neural circuit formation. Mutations in the *CLN8* gene can impair dendritic arborization, leading to widespread neuronal dysfunction with disrupted neuronal communication, contributing to progressive neurodegeneration observed in CLN8 disease [6] and thus to the deterioration of cognitive abilities [7,8]. Indeed, cognitive decline is a hallmark of CLN8 disease, typically manifesting as progressive memory loss, learning difficulties, and intellectual regression [9,10]. These symptoms often start in childhood and worsen over time, following the neurodegeneration observed in the brain, particularly in the cerebral cortex and hippocampus. In addition, patients with CLN8 and other NCLs also present other clinical manifestations such as social deficits and behavioral problems, including aggressive symptoms and anxiety. Children with CLN8 disease or other NCLs can indeed exhibit difficulties in social interactions, including reduced ability to communicate and engage in social play [11,12]. Behavioral problems worsen over time when psychiatric manifestations like aggressive attacks, fear, mood disorders, and psychosis could become a challenge for families and clinicians [9]. The neurodegeneration associated with CLN8 affects brain regions involved in emotional regulation, social cognition, and executive function, contributing to the complex clinical picture of this disease [6]. Overall, it is reasonable to think that the cognitive and social impairments in CLN8 disease are a direct consequence of the underlying neurodegeneration driven by lysosomal dysfunction and abnormal neuronal development. For this reason, targeting lysosomal dysfunction should not only mitigate disease progression but also improve cognitive and social functions, potentially improving the quality of life of patients and their families.

In the past few years, trehalose, a non-reducing disaccharide, has emerged as a substance capable of stimulating autophagy and improving lysosomal function [13], features impaired in CLN8 disease. Trehalose, indeed, enhances autophagy, helping to clear toxic aggregates from cells [14], to stabilize proteins under stress, and to counteract neuronal damage [15]. Several studies demonstrated that trehalose could improve different abilities in neurodegenerative diseases. Recently our group showed that trehalose can improve locomotor performances in *cln8^−/−^* zebrafish mutant larvae by modulating protein expression of some autophagic markers [4]. In the same way, it has also shown potential in the treatment of different forms of neurodegenerative lysosomal storage disorders. Multiple experimental findings have shown that trehalose can mitigate disease features in Lafora disease [14,16] and other NCL models [14,17,18] and that it has neuroprotective properties preventing or slowing the progression of conditions like Alzheimer’s disease and other forms of dementia [19]. In addition, trehalose has been found to impact social behavior positively, possibly by mitigating inflammation and neuroinflammation associated with stress and social isolation [20]. Improvement in the autophagy pathway and enhancement of cognitive outcomes with trehalose were also found in aged and injured mice [21].

One of the most valuable experimental models in biology and translational medicine today is the zebrafish (*Danio rerio*). Sharing lots of genetic, structural, and physiological similarities with humans, zebrafish have been used for studying the pathomechanisms of NCLs [4,22,23,24] and for drug discovery [4]. The primary advantage of zebrafish models lies in their capacity to address complex biological questions and to provide insights into cellular processes that drive disease within a relatively simple organism that shares high synteny and sequence identity with the human genome.

Based on this evidence, the main objective of this study was to verify the possible efficacy of trehalose dietary supplementation on behavioral functions in an adult zebrafish model of CLN8. For this purpose, we explored a battery of motor, cognitive, emotional, and social tasks in disease models by means of standardized behavioral tests to verify the efficacy of trehalose administration to attenuate the observed deficits.

## 2. Materials and Methods

### 2.1. Zebrafish Maintenance

The experiments were conducted on 22 *cln8^−/−^* and 22 wild-type AB (WT) adult zebrafish (6-month-old, 11 males and 11 females per genotype) maintained at the animal facility of the Department of Veterinary Sciences of the University of Pisa. Fish were housed in a recirculating aquaculture system (RAS) equipped with 3.5 L tanks (Tecniplast S.p.A., Buguggiate, Italy), water temperature was set at 28.5 °C, and a 14 h light/10 h dark cycle was applied, according to standard procedures [25,26]. The trial was carried out in accordance with the European Union’s Directive 2010/63/EU on the protection of animals used for scientific purposes, under the supervision of the University of Pisa Animal Care and Use Committee, and in compliance with the 3R principles. The generation of the mutant, using CRISPR/Cas9 technology, was performed with the ethical approval of the Italian Ministry of Health (approval n° 584/2019-PR) as described in our previous study [4].

### 2.2. Dietary Treatment

Before the start of the experimental trial, adult fish were fed with a commercial dry feed formulated for the species (Zebrafeed, Sparos, Olhão, Portugal) 4 times per day [27]. Trehalose (Merck-Millipore, Milan, Italy) supplementation was carried out by including the compound in the pelleted feed (inclusion rate 1% on a fed basis) according to Royes and Chapman [28]. Briefly, the dry feed was first finely ground, then osmosis water (in which trehalose was dissolved) was added to form a dough, and finally the feed was re-pelleted, dried, and stored in the refrigerator until use. Studies in which trehalose was included in the diet have used 1% inclusion rates, demonstrating the safety and efficacy of this concentration [29]. In zebrafish, similar dietary supplementation strategies suggest that 1% is an appropriate concentration for assessing metabolic and behavioral effects [30]. The experimental feeding trial lasted 14 days, and the ordinary feeding management was maintained.

### 2.3. Behavioral Tests

**Figure 1:**
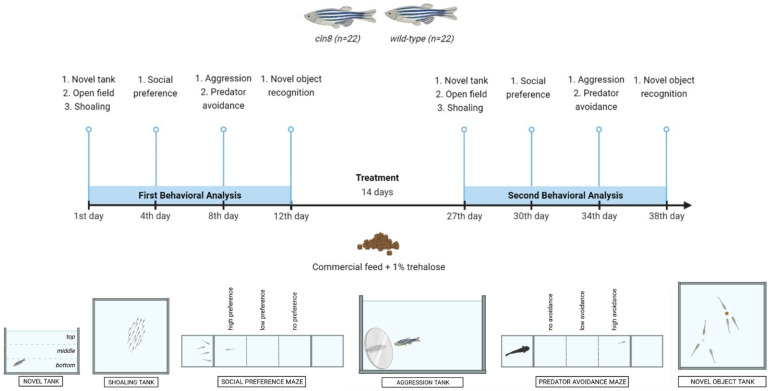
Schematic representation of the experimental trial.

A series of standardized behavioral tests were used to assess various aspects of anxiety, social interaction, aggression, fear, and cognitive function in the zebrafish mutant and WT lines (before and after the feeding supplementation). All tests were performed in tanks filled with RAS water maintained at a constant temperature of 28.5 °C by using a heating mat and under controlled lighting conditions. The animals were tested after their usual morning feeding to prevent stress from food deprivation. The tanks of fish to be tested were kept for two hours in the test room to allow the animals to acclimatize. Each test was repeated after 26 days. Due to the limited availability of mutant fish, it was not feasible to create separate experimental groups (e.g., treated and untreated). To ensure robustness in our statistical analyses and make the best use of our resources, we adopted a paired design, evaluating the same animals before and after trehalose treatment. The schematic representation of the trehalose treatment with the behavioral tests performed is sketched in Figure 1.

In detail, for the novel tank and the aggression tests, a 1.7 L tank (10.6 cm high × 20 cm wide × 12.5 cm long; Tecniplast S.p.A., Buguggiate, Italy) was used, and for the shoaling and the novel object recognition tests, an open-field arena with 40 L of water (40 cm high × 40 cm wide × 40 cm long; Noldus Information Technology, Wageningen, The Netherlands) was employed. Social preference and predator avoidance tests were performed using 5 compartments of a Noldus T-maze (4 L). Each compartment is a transparent cube of 10 cm where it is possible to insert transparent dividers.

#### 2.3.1. Novel Tank Test

The Novel Tank Test was conducted to evaluate the individual’s response to a novel environment, where fish typically exhibit diving behavior by spending more time in the lower section of the tank due to their natural tendency for negative geotaxis when anxious [28,31]. Single zebrafish were introduced into a novel tank, and behavior was video-recorded over a 5 min observation period. The tank was virtually divided into three horizontal zones: bottom, middle, and top. Distance moved, velocity, and time spent in each zone were measured for each tested fish to evaluate their swimming activity and zone preference in an unfamiliar place.

#### 2.3.2. Shoaling Test

The Shoaling Test was employed for assessing social interactions and group dynamics. The advantages of shoaling include protection from predation, which is assumed to increase with increasing shoal density [32]. Increased shoal cohesion and body contacts were “red flags” indicating stronger sociality and comfort within the group, while a decrease of the same behaviors may reflect stress or threat perception [33]. Twenty fish per group were introduced into an open-field arena and allowed to acclimate for 5 min. Then, fish were allowed to explore the tank freely for a period of 10 min. Behavioral parameters measured included total distance to travel, swimming velocity, inter-fish distance, and the number of body contacts. These metrics provided insight into anxiety-related changes in group cohesion and social interaction.

#### 2.3.3. Social Preference Test

The Social Preference Test enables the assessment of fish sociality through the observation of their interactions with conspecifics [34]. Each tested fish was placed in the central compartment of a T-maze (Noldus Information Technology) and allowed to explore the new environment for 5 min. Subsequently, in one of the two side compartments was positioned a group of 3–4 zebrafish of the same age and size (social stimulus), while the other was left empty (no social stimulus). The time spent near the compartment with the social stimulus compared to the empty compartment was recorded for an additional 5 min. An increased duration spent near the compartment with conspecifics indicates a preference for social interaction, while a preference for the empty side suggests individual social impairment. The compartment containing the social stimulus was alternated with the empty one between one test and another to avoid behavioral bias.

#### 2.3.4. Aggression Test

The Aggression Test evaluates the number of bites directed at the mirror as a measure of aggressive behavior, with a focus on distinguishing between male and female aggression patterns. Indeed, zebrafish perceive their reflection as another conspecific (and males as an invading rival), so this test is a standard method for assessing aggression levels in fish [35]. Each zebrafish was introduced into a tank with a mirror positioned at one side. The fish was allowed to acclimate for 5 min, after which its behavior in response to its own reflection was recorded for an additional 5 min. Since aggression can be modulated by anxiety, neurodegeneration, and altered social cognition, this test served as a measure of both social dominance and impulsive aggressive behaviors.

#### 2.3.5. Predator Avoidance Test

The Predator Avoidance Test allows us to measure the innate fear response of the fish to a living predator to evaluate the overall anxiety levels in the presence of a potential lethal risk [35]. Predator avoidance is a fundamental survival behavior, and deficits in this response may indicate alterations in innate fear processing and anxiety regulation. Single zebrafish were placed in the central test compartment of the T-maze (the same used for the Social Preference Test) and allowed to acclimate for 5 min. Following this acclimation period, a carnivorous larger fish (*Astronotus ocellatus*) was introduced into an adjacent compartment. The behavior of the zebrafish was recorded for an additional 5 min after the introduction of the predator. Measurements included the time spent near the predator compartment (no avoidance area), the one in the middle (low avoidance), and the one furthest from the predator (high avoidance). The compartment containing the living predator was alternated with the empty one between one test and another to avoid behavioral bias.

#### 2.3.6. Novel Object Recognition Test

The Novel Object Recognition Test assesses zebrafish’s exploratory behavior towards novel or familiar objects. Zebrafish are naturally curious and tend to explore novel objects more than familiar ones. Reduced exploration of the novel object can indicate anxiety or impaired cognitive function [36]. The test consists of three phases, each lasting one day. On the first day (habituation day), 12 zebrafish per genotype were acclimated to the new tank without any objects. On the second day (training day), two identical floating objects were introduced into the tank (two aluminum or wooden balls of the same size but different in color and texture), allowing the fish to familiarize themselves with them. On the third day (testing day), the test was carried out, presenting to the fish one familiar object (the same from day two) and a novel floating object (never seen before). The number of interactions with each object is recorded over a 10 min period. This design allows for an evaluation of the fish’s cognitive function and anxiety levels, with a preference for the novel object indicating healthy exploratory behavior.

### 2.4. Statistical Analysis

We analyzed data using parametric or non-parametric methods, based on the distribution of the response variable, which was assessed via the Shapiro–Wilk test. Normally distributed data were analyzed with the unpaired *t*-test, while non-normally distributed data were analyzed with the Mann–Whitney test. Statistical significance was defined as *p* ≤ 0.05, and all analyses were conducted using GraphPad Prism 9 (Graph-Pad Software, San Diego, CA, USA).

## 3. Results

### 3.1. Trehalose Reduces cln8^−/−^ Anxiety-like Behaviors

**Figure 2:**
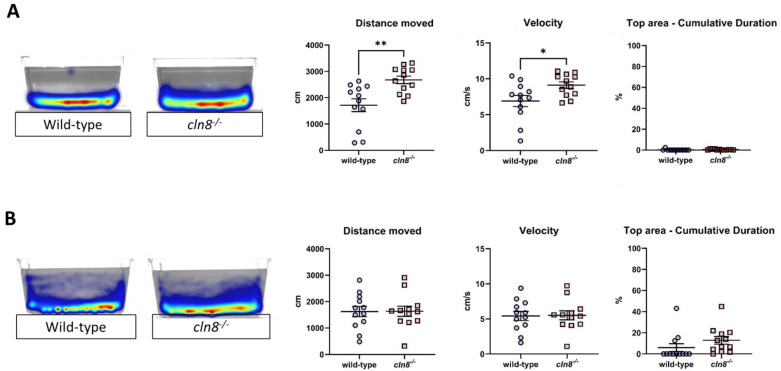
Novel tank test behavioral results before (**A**) and after (**B**) the trehalose treatment on *cln8^−/−^* and WT fish (*n* = 12). Data are represented as individual values (lines indicate means ± SEM). Statistical analyses showed increasing anxiety in *cln8^−/−^* compared to WT before the trehalose feed supplementation (* *p* ≤ 0.05; ** *p* ≤ 0.01). The images on the left are representative of the heatmap locomotor activity during the test, the range of color is from dark blue (low activity) to red (high activity).

The Novel Tank Test results indicated that, prior to trehalose treatment, *cln8^−/−^* mutant fish exhibited significantly higher swimming activity compared to WT fish, suggesting elevated anxiety-like behaviors in response to a novel environment. We interpreted changes in distance moved and velocity as anxiety-related based on established literature that links increased or decreased locomotor activity to anxiety, depending on the context and experimental conditions [37,38]. Although there are additional parameters, such as freezing bouts and erratic movements, we did not include these analyses in order to allow us to standardize the interpretation of our data in relation to anxiety while minimizing potential variability due to experimental or contextual differences. Despite this increased activity, neither group explored the upper area of the tank (Figure 2A), which was unexpected given that zebrafish typically explore the upper area of the tank, although they tend to spend more time at lower depths, especially under certain conditions such as novelty or stress. This deviation from typical zebrafish behavior could potentially be attributed to various factors, including the experimental conditions—such as the novelty of the tank, water depth, or the presence of visual cues—that may have influenced their exploration patterns. It is also possible that perceived environmental stressors or a preference for the more secure lower regions of the tank contributed to their limited use of the upper area. Following trehalose treatment, the behavior of *cln8^−/−^* mutants aligned more closely with that of the WT fish in terms of distance moved and swimming velocity. Specifically, trehalose supplementation appeared to reduce anxiety levels in *cln8^−/−^* mutants, as evidenced by a decrease in swimming distance and speed and an increased exploration of the entire tank, including the top area (Figure 2B and Figure S1B). No difference was observed in WT fish following dietary treatment (Figure S1A).

### 3.2. Trehalose Ameliorates WT and cln8^−/−^ Groups Cohesion

**Figure 3:**
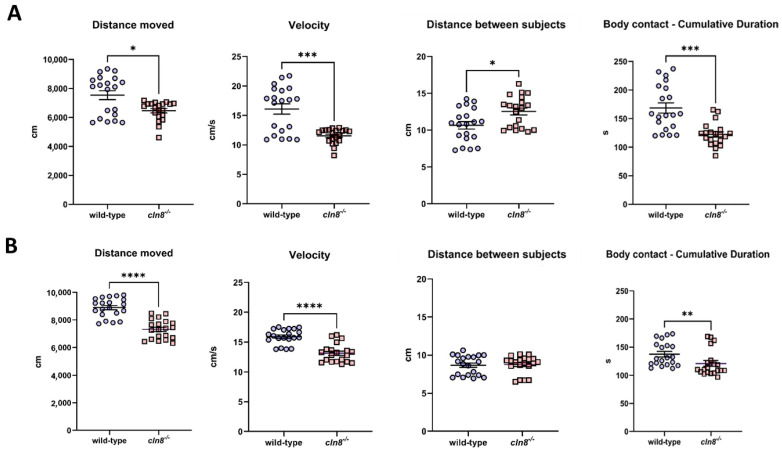
Shoaling test behavioral results before (**A**) and after (**B**) the trehalose treatment on *cln8^−/−^* and WT fish (*n* = 20). Data are represented as individual values (lines indicate means ± SEM). Statistical analyses showed impairments in swimming performances and shoal cohesion in *cln8^−/−^* compared to WT both before and after the trehalose feed supplementation (except for distance between subjects mitigated by trehalose treatment) (* *p* ≤ 0.05; ** *p* ≤ 0.01; *** *p* ≤ 0.001; **** *p* ≤ 0.0001).

The Shoaling Test revealed a persistent locomotor impairment in the *cln8^−/−^* mutant fish compared to the WT group, both before and after trehalose treatment. In this case, fish were already acclimated to the new tank and swam in groups rather than alone, reducing stress-inducing stimuli. Despite these conditions, *cln8^−/−^* mutants exhibited reduced distance moved and lower swimming velocity compared to the WT fish shoal (Figure 3A,B), supporting the idea that their behavior in group settings is significantly altered, possibly due to deficits in social interaction or an inability to synchronize their movements with those of the group. Conversely, the novel tank test is designed to assess exploratory behavior in an unfamiliar environment. In this context, increased movement and speed in *cln8^−/−^* zebrafish could be attributed to heightened anxiety, stress-induced hyperactivity, or an inability to regulate exploratory drive. Such responses are commonly observed in zebrafish with altered neural or behavioral regulation. These findings suggest that the distinct contexts of social versus exploratory paradigms evoke different behavioral patterns in *cln8^−/−^* zebrafish, likely reflecting the diverse neural circuits and mechanisms engaged in each scenario. Regarding shoaling behavior, mutant fish showed an increased inter-individual distance, indicating reduced group cohesion, while WT fish maintained a more compact formation. However, trehalose treatment partially mitigated this deficit, leading to improved shoal compactness in the mutant group (Figure S2B). Overall, dietary supplementation with trehalose increased the swimming activity and the shoal cohesion of both fish groups (Figure S2A,B), highlighting a positive effect of the treatment on social interaction even in a non-stressful context.

### 3.3. Trehalose Slightly Enhances Sociality in WT and cln8^−/−^

**Figure 4:**
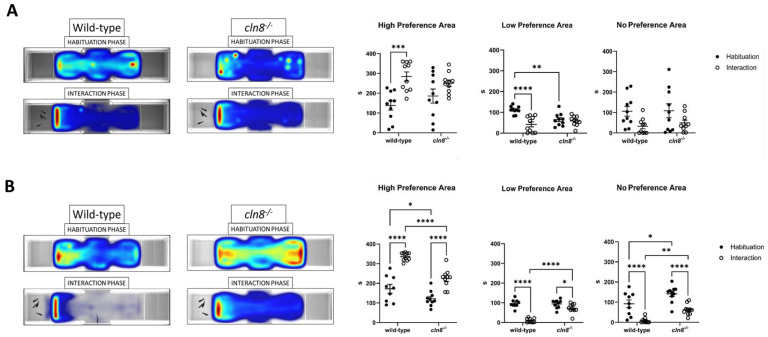
Social preference test behavioral results before (**A**) and after (**B**) the trehalose treatment on *cln8^−/−^* and WT fish (*n* = 10). Data are represented as individual values (lines indicate means ± SEM) and expressed in terms of cumulative duration. Statistical analyses showed impairments in sociality in *cln8^−/−^* compared to WT before the trehalose feed supplementation (* *p* ≤ 0.05; ** *p* ≤ 0.01; *** *p* ≤ 0.001; **** *p* ≤ 0.0001). The images on the left are representative of the heatmap locomotor activity during the test; the range of color is from dark blue (low activity) to red (high activity).

The Social Preference Test showed that, under standard feeding laboratory conditions, *cln8^−/−^* mutant fish demonstrated a weaker preference for conspecifics compared to WT fish. Mutants spent slightly less time in the high-preference area during the interaction phase compared to the habituation period (Figure 4A), suggesting reduced social motivation. Calculation of the social index highlighted this difference, with mutant fish displaying slightly lower social index values compared to WT fish (Supplementary Materials Table S1). While the difference was not statistically significant, it indicates a trend toward reduced social preference in mutants. During the habituation phase, both mutants and WT fish explored the compartments similarly, with *cln8^−/−^* showing no significant preference for the future conspecific area. This suggests that any potential side bias during habituation was minimal, and the reduced social motivation in mutants becomes apparent only during the interaction phase. Indeed, mutants failed to spend more time in the high-preference area during the interaction phase in comparison to the habituation period (Figure 4A), indicating reduced social motivation. After dietary treatment with trehalose, mutant fish showed a significant increase in time spent in the high-preference area during the interaction phase compared to the habituation period, more closely resembling the behavior of WT fish (Figure 4B). These findings suggest that trehalose supplementation slightly enhances social behavior in all fish, with a particularly notable effect on *cln8^−/−^*, who initially displayed weaker social interactions (Figure S3). Behavioral analysis results before and after the dietary treatment for both fish lines are presented in Figure S4.

### 3.4. Trehalose Reduces cln8^−/−^ Aggressiveness

**Figure 5:**
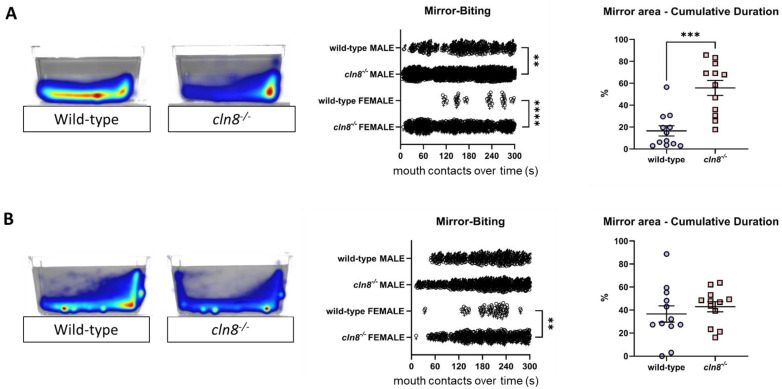
Aggression test results before (**A**) and after (**B**) trehalose treatment on *cln8^−/−^* and WT fish (*n* = 12). Data are represented as individual values (lines indicate means ± SEM). Statistical analyses showed higher aggressiveness in *cln8^−/−^* compared to WT before the trehalose feed supplementation (except for females, which proved to be more aggressive even after treatment) (** *p* ≤ 0.01; *** *p* ≤ 0.001; **** *p* ≤ 0.0001). The images on the left are representative of the heatmap locomotor activity during the test; the range of color is from dark blue (low activity) to red (high activity).

The Aggression Test indicated that *cln8^−/−^* mutant fish exhibited significantly higher levels of aggressive behavior compared to WT fish, in both males and females (Figure 5A). Mutant fish showed a markedly increased frequency of mirror-biting, and notably, even female *cln8^−/−^* fish displayed elevated aggression levels. This intensified aggressiveness was further evidenced by the cumulative time spent close to the mirror, which was approximately three times longer in mutants compared to WT fish. However, after trehalose supplementation, the aggressiveness of *cln8^−/−^* mutants was reduced, with a notable decrease in both the frequency of mirror-biting and the time spent near the mirror, particularly in males (Figure S5). Although female *cln8^−/−^* mutants continued to exhibit higher aggression levels compared to WT females (Figure 5B), the dietary treatment still led to a significant reduction in the number of mirror-biting events (Figure S5).

### 3.5. Trehalose Rescued Fear Response in cln8^−/−^

**Figure 6:**
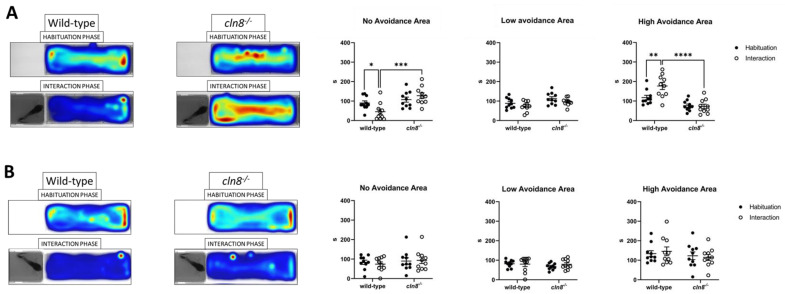
Predator avoidance test results before (**A**) and after (**B**) trehalose treatment on *cln8^−/−^* and WT fish (*n* = 10). Data are represented as individual values (lines indicate means ± SEM) and expressed in terms of cumulative duration. Statistical analyses showed impairments in predator avoidance in *cln8^−/−^* compared to WT before the trehalose feed supplementation (* *p* ≤ 0.05; ** *p* ≤ 0.01; *** *p* ≤ 0.001; **** *p* ≤ 0.0001). The images on the left are representative of the heatmap locomotor activity during the test; the range of color is from dark blue (low activity) to red (high activity).

Results of the Predator Avoidance Test demonstrated that *cln8^−/−^* mutant fish, prior to trehalose treatment, spent significantly more time in the no-avoidance area compared to wild-type (WT) fish, which preferred the high-avoidance area (Figure 6A). In the first test, it appeared that the mutants did not instinctively recognize the bigger unknown fish, as they frequently approached the boundary separating them from the predator. This behavior was partially rescued following trehalose supplementation, as *cln8^−/−^* fish exhibited avoidance patterns more similar to those of WT fish, actively avoiding the area near the predator (Figure 6B, Figure S6, and Figure S7). Behavioral analysis results before and after the trehalose treatment for both fish lines are presented in Figure S7.

### 3.6. Trehalose Has Only a Mild Effect on Cognition in cln8^−/−^ Models

**Figure 7:**
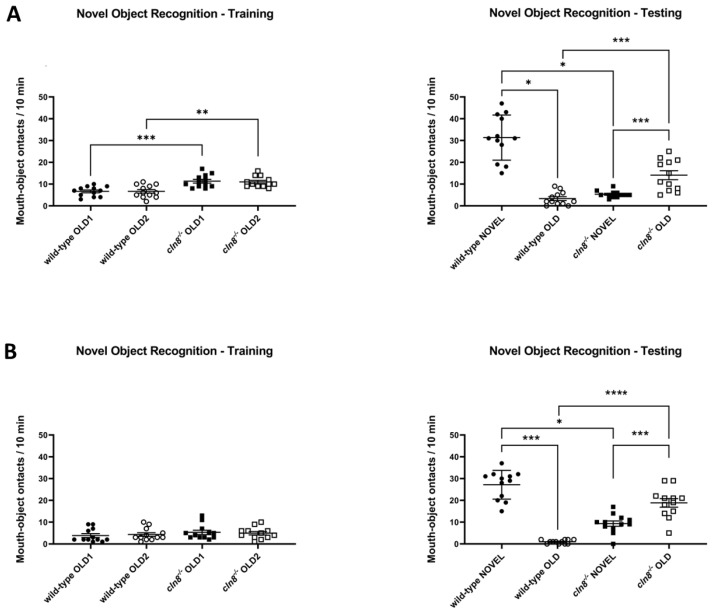
Novel object tests behavioral results before (**A**) and after (**B**) the trehalose treatment on *cln8^−/−^* and WT fish (*n* = 12). Data are represented as individual values (lines indicate means ± SEM). Statistical analyses showed impairments in cognitive abilities in *cln8^−/−^* compared to WT both before and after the trehalose feed supplementation (* *p* ≤ 0.05; ** *p* ≤ 0.01; *** *p* ≤ 0.001; **** *p* ≤ 0.0001).

The results of the novel object test confirmed the cognitive dysfunction observed in *cln8^−/−^* mutant fish, as they showed increasing biting behaviors in the training phase and a lack of preference for interacting with the novel object during the test compared to wild-type (WT) fish, which exhibited significantly more biting behavior toward the new object (Figure 7A). Notably, trehalose treatment did not alter this behavioral pattern, and the differences between mutants and WT fish persisted. However, during the training, *cln8^−/−^* mouth-contacts with familiar objects were similar to control fish, and interactions with the novel object were increased (practically doubled) compared to their pre-treatment behavior (Figure 7B), suggesting a positive significant effect on cognition (Figure S8).

## 4. Discussion

Cognitive decline, emotional problems, and mild social deficits are frequent manifestations in patients with CLN8 and other NCL types, being features that typically worsen as the disease progresses [12,39,40]. Previous findings highlighted the role of CLN8 in maintaining lysosomal pH and the autophagic process and ensuring proper dendritic arborization, a crucial factor for synaptic function and neural circuit formation [6]. Cognitive, emotional, and social dysfunctions observed in patients with *CLN8* mutations are direct consequences of the impaired neuronal function and worsen with the progression of the neurodegeneration [7,8].

In this study, we investigated motor, emotional, social, and cognitive abilities in an adult zebrafish model of CLN8, revealing notable impairments in locomotion, cognition, aggression, anxiety, and social interactions. A comprehensive characterization of these features has not been previously explored in mice. This evidence underscores the utility of zebrafish as a valuable model for assessing the potential therapeutic effects of various compounds on the cognitive and emotional dimensions of CLN8 disease, which significantly impact the quality of life of affected patients and their families. Trehalose is a known neuroprotective agent able to enhance autophagy and facilitate the clearance of toxic cellular aggregates [14,17]. Moreover, trehalose has been shown to confer sustained neuroprotective effects and promote autophagy in mouse models of Huntington’s disease and tauopathies when administered over extended periods [41,42]. These findings suggest that trehalose could potentially maintain its therapeutic efficacy with prolonged use, but further investigations are required to confirm this in CLN8-associated NCLs. Notably, trehalose is known to induce autophagy by inhibiting mTORC1 signaling, which leads to the activation of the autophagic pathway [12]. Additionally, trehalose has been shown to stimulate lysosomal biogenesis through activation of TFEB, a master regulator of lysosomal and autophagic genes [43,44]. These studies suggest that trehalose’s therapeutic effects may be mediated through its capacity to enhance autophagy and support cellular homeostasis by regulating lysosomal function. Our previous research has demonstrated that trehalose improves autophagy in *cln8^−/−^*-treated larvae [4], and it has also been shown to reduce oxidative stress and inflammation, both critical contributors to neurodegenerative diseases [19,45]. Alterations in social behavior have been reported in CLN8 mouse models, encompassing motor impairments and cognitive deficits that negatively affect social interactions. For instance, *mnd/mnd* mice, a faithful model of CLN8, exhibit neurological symptoms from an early age, including pronounced behavioral changes [46]. Moreover, Inoue et al. [47] showed that some patients with rare missense mutations in *CLN8* were diagnosed with autism spectrum disorder, suggesting a potential link between CLN8 dysfunction and alterations in social behaviors observed in autism. Numerous studies utilizing cellular and animal models have investigated the efficacy of trehalose in addressing the NCL phenotype, demonstrating improvements in motor impairments and seizures [14,16,17]. In this study, we specifically focused on the potential impact of trehalose on cognitive and emotional features. Interestingly, trehalose effectively ameliorated various emotional parameters, including aggression, fear, and anxiety, alongside a modest influence on social behaviors in adult mutant zebrafish. This highlights its neuroprotective effects and suggests its potential to modulate behavioral responses that are impaired by the disease. These findings align with preliminary evidence that emerged in an open-label clinical trial in NCL where the caregiver perspective after treatment suggested improved mood stability, increased participation in daily activities, and enhanced environmental engagement [48]. However, future studies could focus on conducting detailed morphological analyses (e.g., histological or imaging studies) to assess structural changes in tissues or organs following trehalose treatment. Cellular investigations into pathways such as autophagy or apoptosis could help clarify the therapeutic mechanisms involved. Additionally, transcriptomic or proteomic evaluations may reveal changes in gene or protein expression profiles that correlate with the observed behavioral and therapeutic outcomes. Incorporating these approaches could provide a more comprehensive understanding of trehalose’s effects and its potential applications in treating the disease modeled in zebrafish. Furthermore, investigating the effects of trehalose across a range of neurodegenerative diseases could offer valuable insights into its broader therapeutic potential. It is essential to determine whether trehalose’s neuroprotective actions are specific to particular disease mechanisms or if they provide a more generalized approach to managing common features such as protein misfolding and lysosomal dysfunction, which are implicated in several neurodegenerative disorders. Such effects could potentially extend to other variants of NCLs as well.

## 5. Conclusions

Overall, the findings presented in this study establish a robust framework for both modeling the cognitive and emotional manifestations of NCLs and identifying potential therapeutic agents that may alleviate these disease-related features. Specifically, our results show that trehalose has a positive impact on emotional and mild social deficits in *cln8^−/−^* mutant zebrafish, improving the level of anxiety, sociability, and predator avoidance. These findings suggest that trehalose may alleviate some of the debilitating symptoms associated with CLN8 disease, with the potential to improve the quality of life in affected individuals. The mechanistic outlook of these events remains yet unsolved, but it could lay into the modulation of cellular autophagy pathways, given trehalose’s known role as an autophagy enhancer, or stimulation of lysosomal biogenesis. Further investigation into the molecular mechanisms underlying trehalose’s effects on neuronal resilience and synaptic function may provide deeper insights into its therapeutic potential. Additionally, future studies should focus not only on investigating the long-term effects of trehalose treatment and whether its benefits extend to other aspects of neurodegeneration in CLN8-related NCL but also on how to translate these findings in NCL children.

## Data Availability

The data are available from the authors upon reasonable request.

## Supplementary Materials

The following supporting information can be downloaded at: https://www.mdpi.com/article/10.3390/cells14010055/s1, Figure S1: Novel tank test behavioral results before (Pre-treatment) and after the trehalose treatment (Pre-treatment) on WT fish (A) and *cln8*^−/−^ (B) (*n* = 12). Figure S2: Shoaling test behavioral results before (Pre-treatment) and after the trehalose treatment (Pre-treatment) on WT fish (A) and *cln8*^−/−^ (B) (*n* = 20). Figure S3: Social preference test behavioral results before (A) and after (B) the trehalose treatment on WT and *cln8*^−/−^ fish (*n* = 10). Figure S4: Social preference test behavioral results before (Pre-treatment) and after the trehalose treatment (Pre-treatment) on WT fish (A) and *cln8*^−/−^ (B) (*n* = 10). Figure S5: Aggression test behavioral results before (Pre-treatment) and after the trehalose treatment (Pre-treatment) on WT fish (A) and *cln8*^−/−^ (B) (*n* = 12). Figure S6: Predator avoidance test behavioral results before (Pre-treatment) and after the trehalose treatment (Pre-treatment) on WT fish (A) and *cln8*^−/−^ (B) (*n* = 10). Figure S7: Predator avoidance test behavioral results before (A) and after (B) the trehalose treatment on WT and *cln8*^−/−^ fish (*n* = 10). Figure S8: Novel object recognition test behavioral results before (Pre-treatment) and after the trehalose treatment (Pre-treatment) on WT fish (A) and *cln8*^−/−^ (B) (*n* = 12). Table S1: Social index interaction phase – Pre-treatment (*p* = 0.5639). Table S2: *cln8*^−/−^ cognition index testing phase—Pre-treatment vs. post-treatment (*p* = 0.7057).
