# Supplementary material for: Trehalose Ameliorates Zebrafish Emotional and Social Deficits Caused by CLN8 Dysfunction
Source: Cells. 2025 Jan 5;14(1):55. doi: 10.3390/cells14010055 (PMC11720655; doi:10.3390/cells14010055)
Supplement: Supplementary file 1 [file cells-14-00055-s001.zip › cells-3330749-supplementary.pdf]

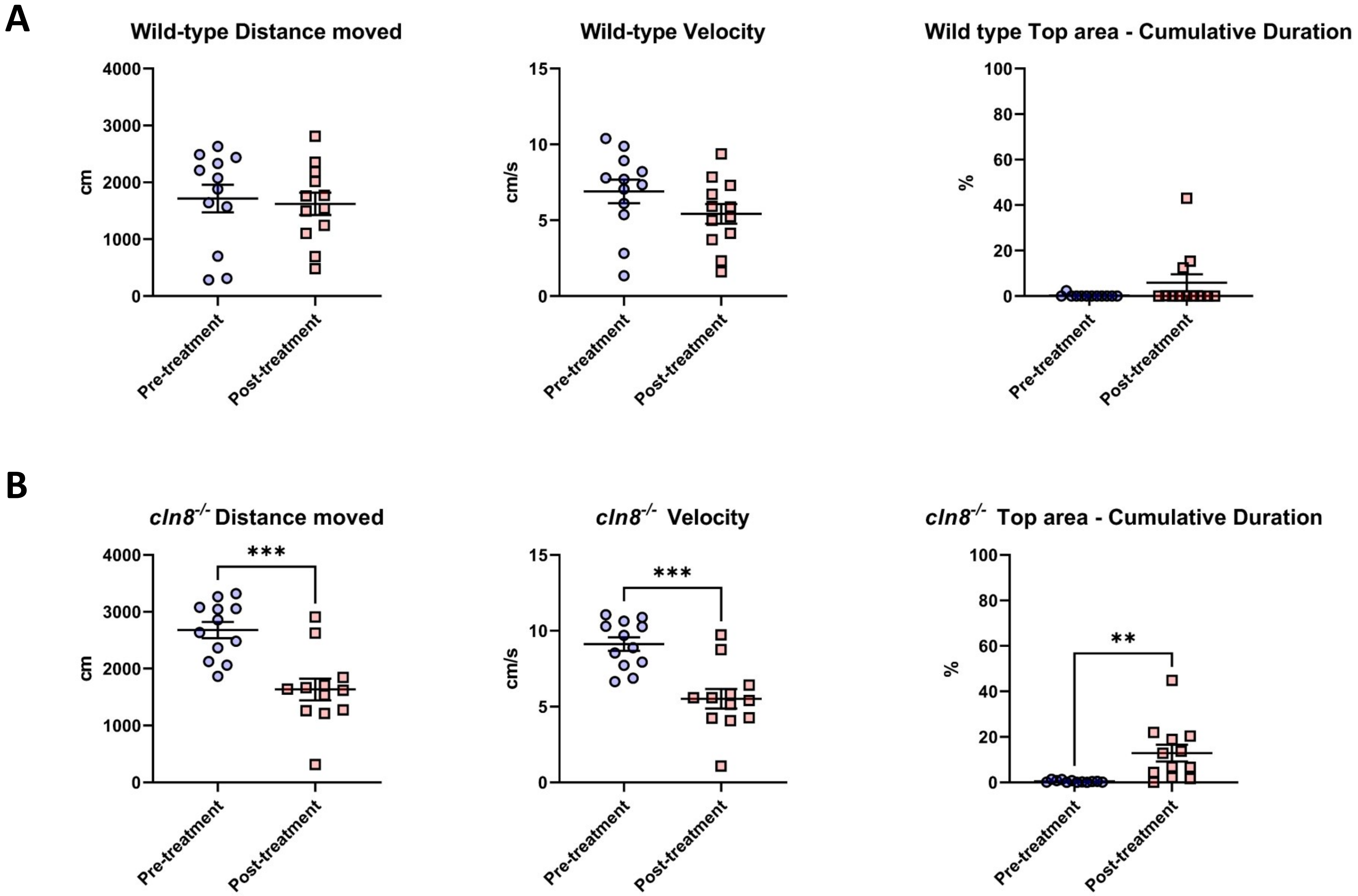

Figure S1. Novel tank test behavioral results before (Pre-treatment) and after the trehalose treatment (Pre-treatment) on WT fish (**A**) and *cln8*$^{-/-}$ (**B**) (n=12). Data are represented as individual values (lines indicate means ± SEM). Statistical analyses showed reduced anxiety in *cln8*$^{-/-}$ after the trehalose feed supplementation (**$p \leq 0.01$; ***$p \leq 0.001$).

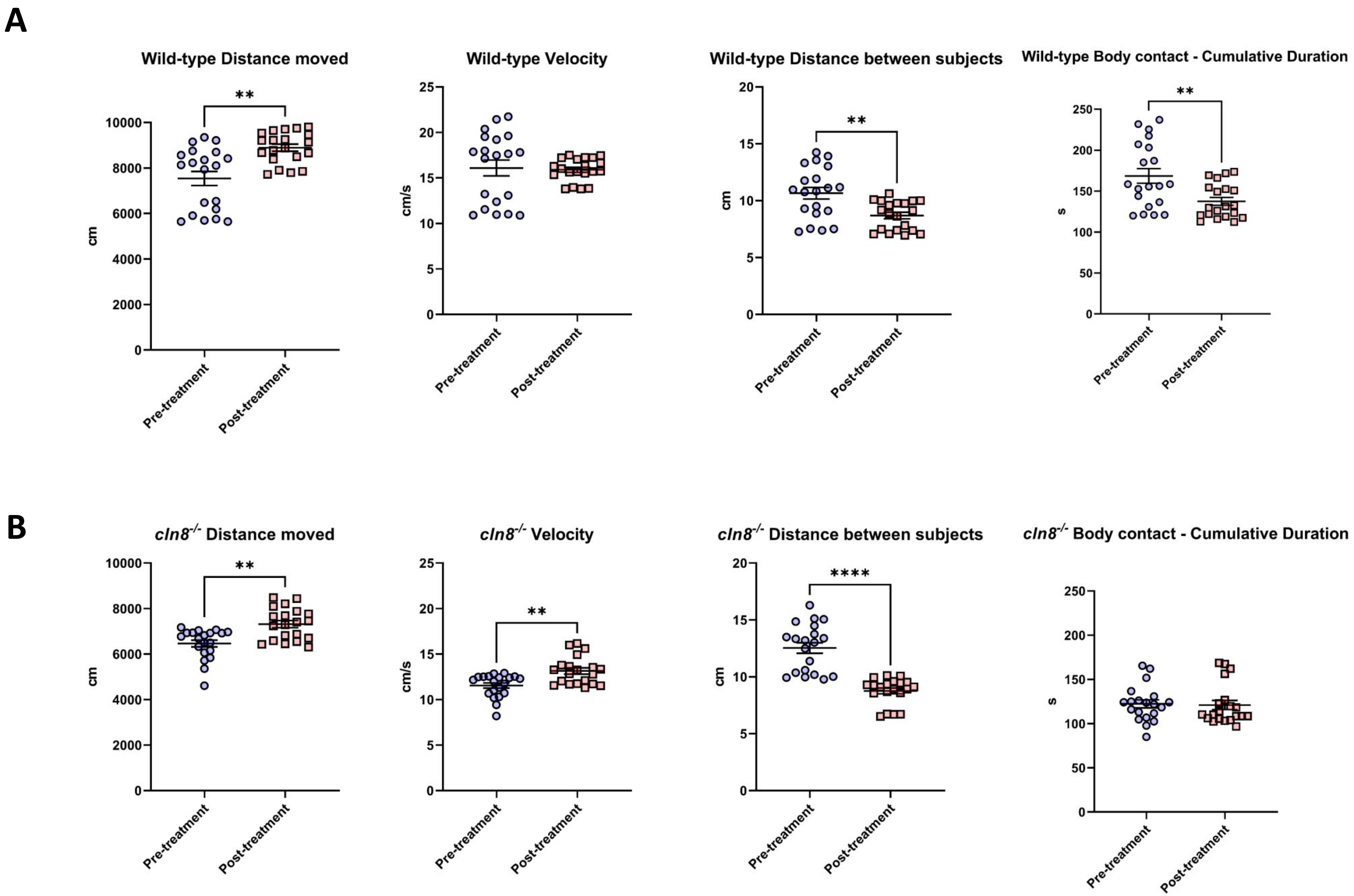

Figure S2. Shoaling test behavioral results before (Pre-treatment) and after the trehalose treatment (Pre-treatment) on WT fish (**A**) and *cln8*$^{-/-}$ (**B**) (n=20). Data are represented as individual values (lines indicate means ± SEM). Statistical analyses showed improved shoal swimming performances and subjects' cohesion in both WT and *cln8*$^{-/-}$ after the trehalose feed supplementation (**$p \leq 0.01$; ****$p \leq 0.0001$).

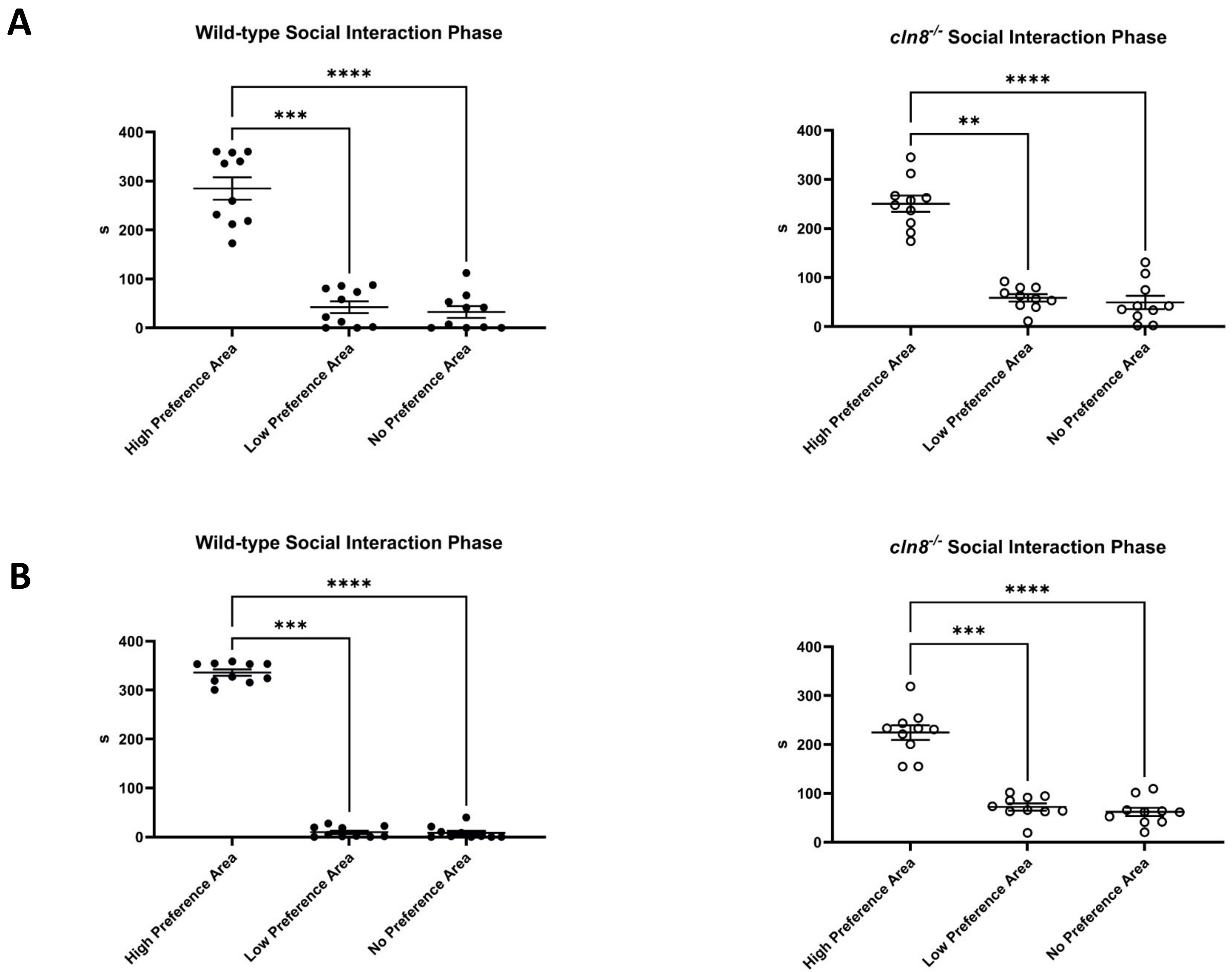

Figure S3. Social preference test behavioral results before (**A**) and after (**B**) the trehalose treatment on WT and *cln8*$^{-/-}$ fish (n=10). Data are represented as individual values (lines indicate means $\pm$ SEM) and expressed in terms of cumulative duration. Statistical analyses showed that differences between high and low preference area in *cln8*$^{-/-}$ were higher after the trehalose feed supplementation (**$p \leq 0.01$; ***$p \leq 0.001$; ****$p \leq 0.0001$).

A

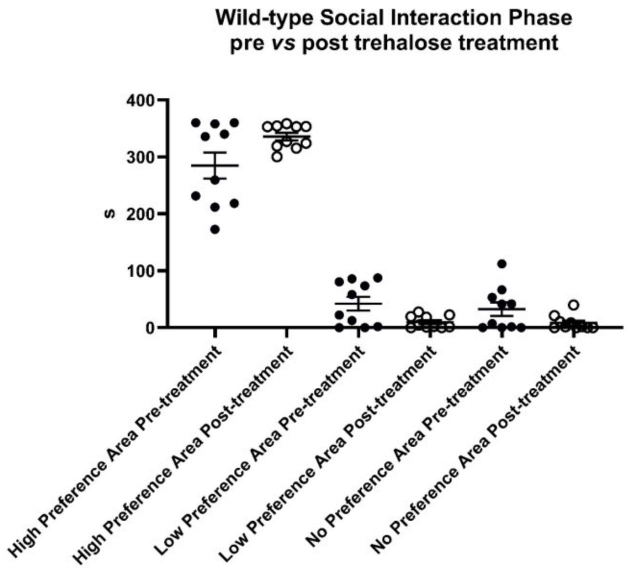

B

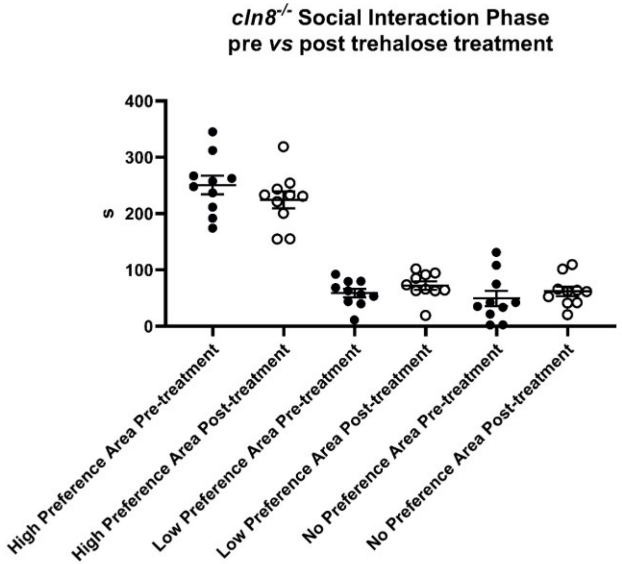

Figure S4. Social preference test behavioral results before (Pre-treatment) and after the trehalose treatment (Pre-treatment) on WT fish (**A**) and *cln8*$^{-/-}$ (**B**) (n=10). Data are represented as individual values (lines indicate means ± SEM). Statistical analyses showed no significant effects in both WT and *cln8*$^{-/-}$ after the trehalose feed supplementation when only the social interaction phase is evaluated.

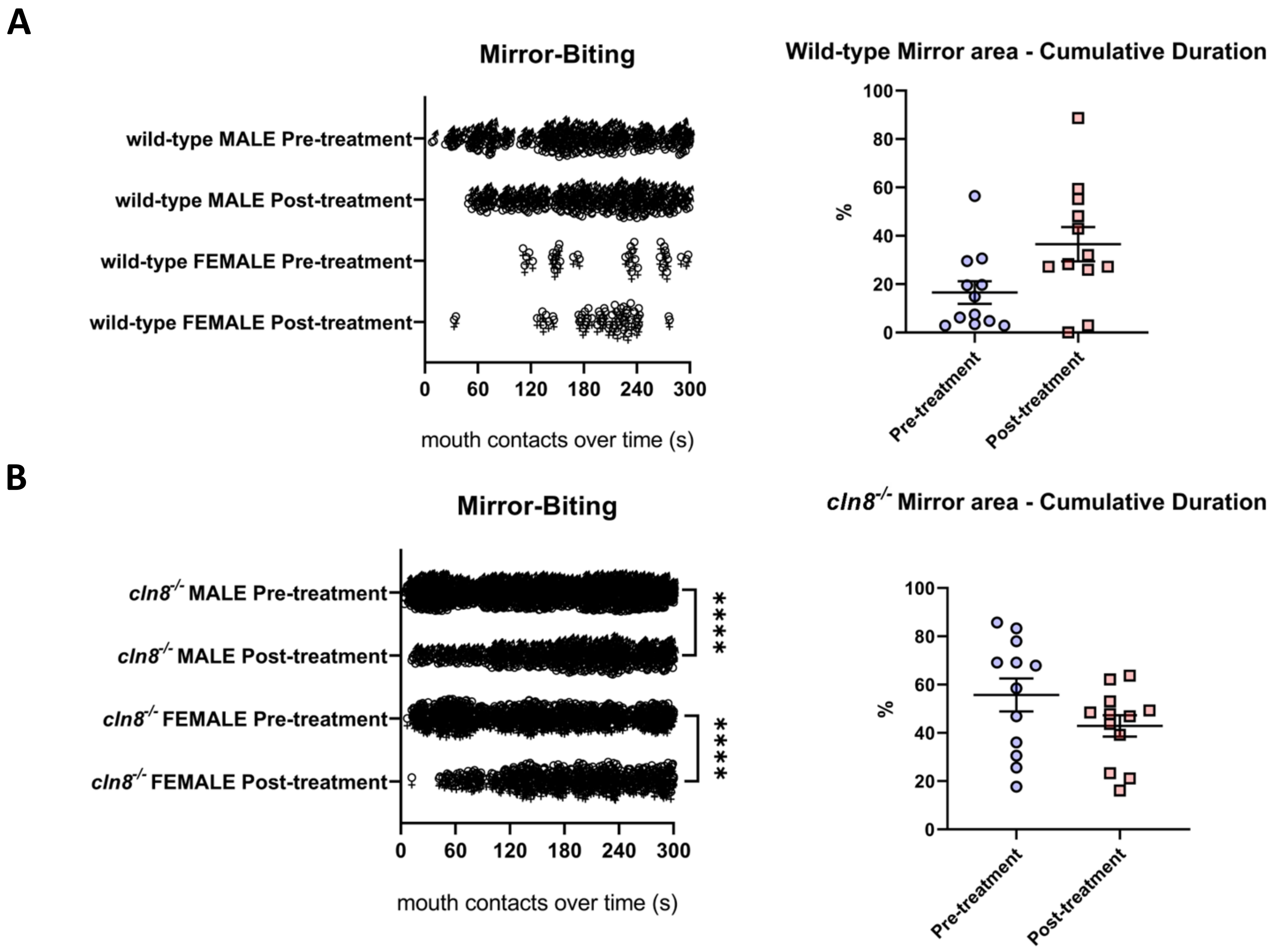

Figure S5. Aggression test behavioral results before (Pre-treatment) and after the trehalose treatment (Pre-treatment) on WT fish (**A**) and *cln8*$^{-/-}$ (**B**) (n=12). Data are represented as individual values (lines indicate means ± SEM). Statistical analyses showed reduced aggressiveness in *cln8*$^{-/-}$ after the trehalose feed supplementation in both males and females (*****p* ≤ 0.0001).

A

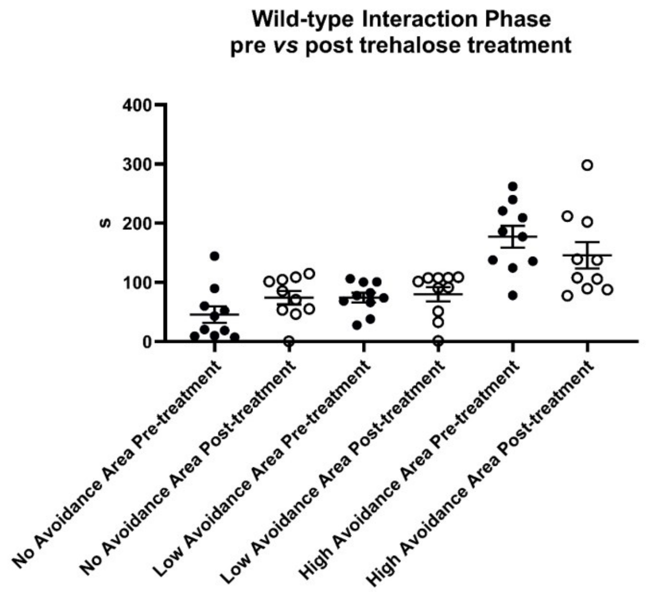

B

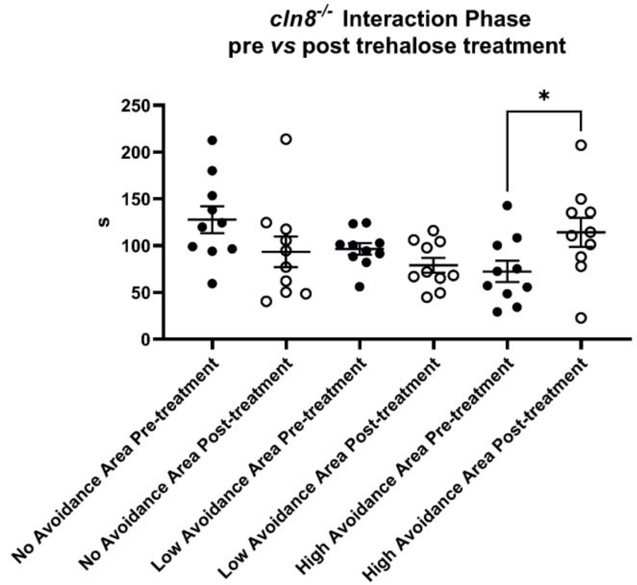

Figure S6. Predator avoidance test behavioral results before (Pre-treatment) and after the trehalose treatment (Pre-treatment) on WT fish (**A**) and *cln8*$^{-/-}$ (**B**) (n=10). Data are represented as individual values (lines indicate means ± SEM). Statistical analyses showed improved predator avoidance in *cln8*$^{-/-}$ after the trehalose feed supplementation when only the predator interaction phase is evaluated.

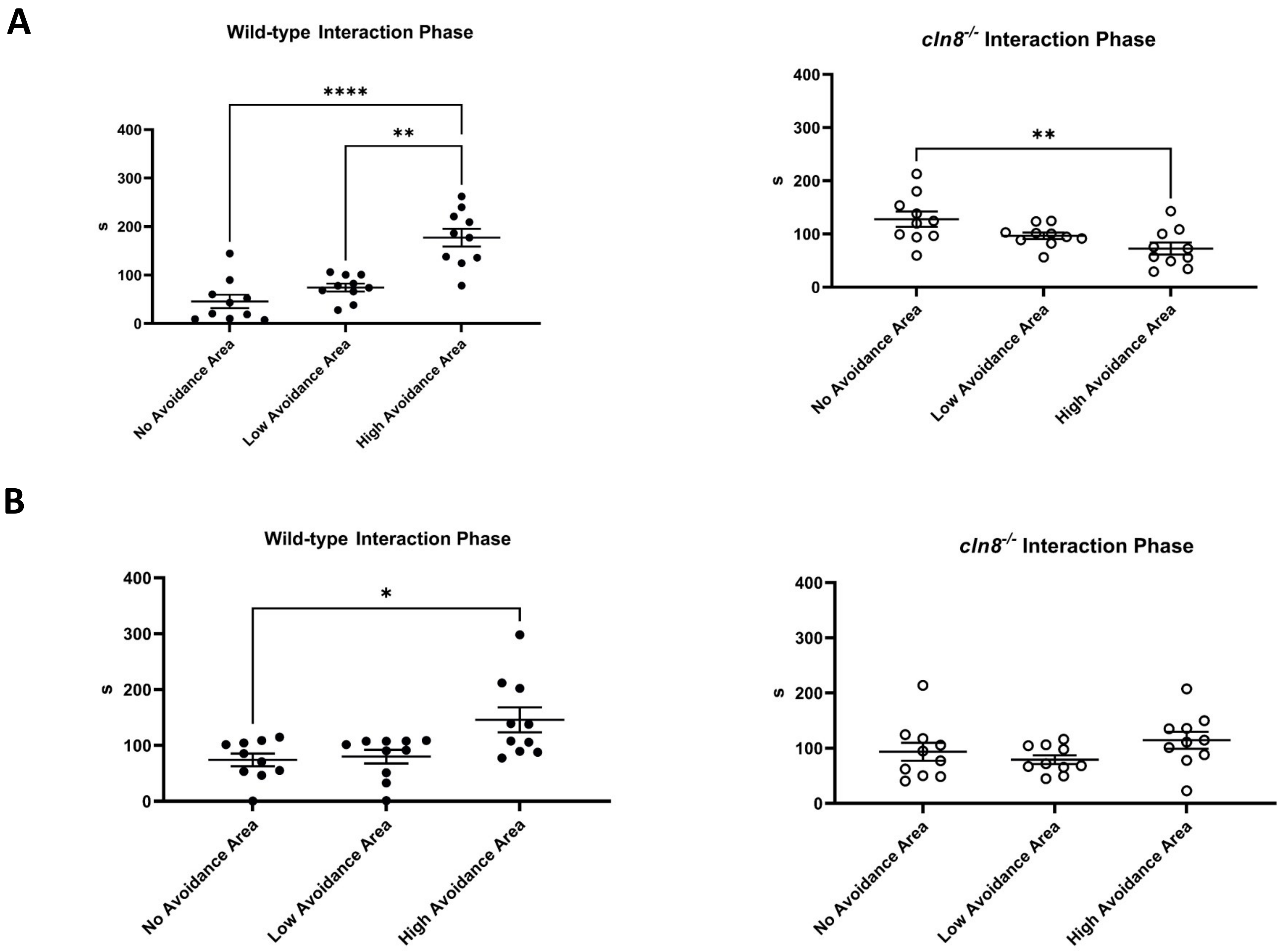

Figure S7. Predator avoidance test behavioral results before (**A**) and after (**B**) the trehalose treatment on WT and *cln8*$^{-/-}$ fish (n=10). Data are represented as individual values (lines indicate means ± SEM) and expressed in terms of cumulative duration. Statistical analyses showed that *cln8*$^{-/-}$ reduce their preference for the No Avoidance Area after the trehalose feed supplementation (*$p \leq 0.05$; **$p \leq 0.01$; ****$p \leq 0.0001$).

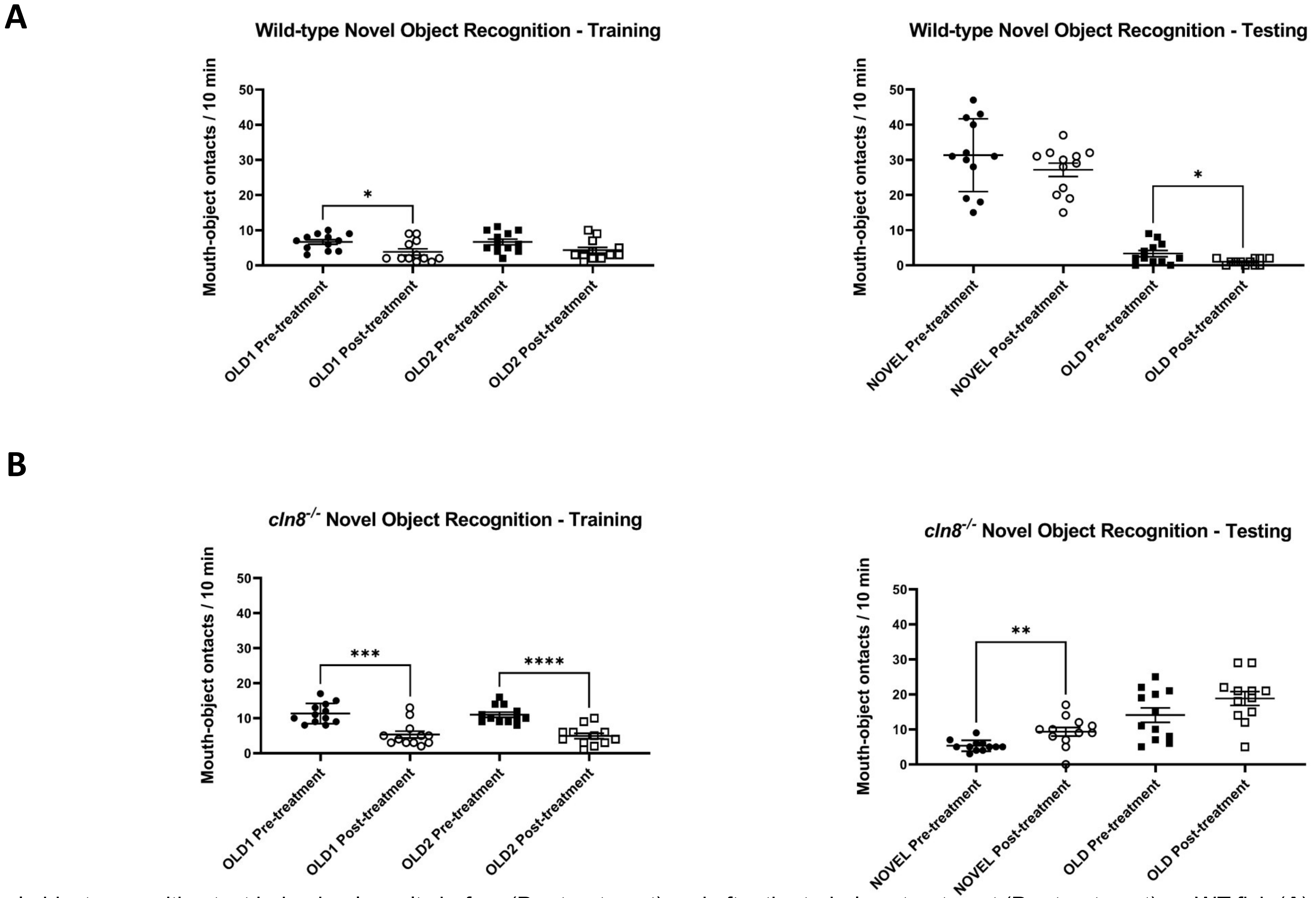

Figure S8. Novel object recognition test behavioral results before (Pre-treatment) and after the trehalose treatment (Pre-treatment) on WT fish (**A**) and *cln8*$^{-/-}$ (**B**) (n=12). Data are represented as individual values (lines indicate means ± SEM). Statistical analyses showed improved cognition in *cln8*$^{-/-}$ after the trehalose feed supplementation (*$p \leq 0.05$; **$p \leq 0.01$; ***$p \leq 0.001$; ****$p \leq 0.0001$).

	**wild-type**	***cln8***$^{-/-}$
	0,929673	0,532483
	1	0,497051
	1	0,527075
	0,989816	0,753253
	0,875302	0,650454
	0,448497	0,682187
	0,428385	0,566674
	0,404089	0,612824
	0,459533	0,937705
	0,634113	0,35054
Mean	0.7169	0.6110
Std. Deviation	0.2649	0.1597
Std. Error of mean	0.08376	0.05051

Tab S1. Social index interaction phase – Pre-treatment (p=0.5639)

Social index = [(Thigh-Tlow)/ (Thigh+Tlow)]

Thigh: time in high preference area

Tlow: time in low preference area

	cln8$^{-/-}$ **pre-treatment**	***cln8***$^{-/-}$ **post-treatment**
	0,076923	0,166667
	-0,57895	-0,27273
	-0,61538	-0,38462
	0,285714	-0,52632
	-0,16667	-0,10526
	-0,375	-0,03448
	-0,72727	-0,09091
	-0,66667	-0,45
	-0,57143	-0,375
	-0,25	-0,47368
Mean	-0,37384	-0,32529
Std. Deviation	0,323727	0,297401
Std. Error of mean	0,102371	0,094046

Tab S2. *cln8*$^{-/-}$ cognition index testing phase – Pre-treatment vs post-treatment (p=0.7057)

Cognition index = [(Cnovel-Cfamiliar)/(Cnovel+Cfamiliar)]

Cnovel: Contacts on the novel object

Cfamiliar: Contacts on the familiar object
